# Colony morphotype diversification as a signature of bacterial evolution

**DOI:** 10.1093/femsml/uqad041

**Published:** 2023-10-10

**Authors:** Ákos T Kovács

**Affiliations:** Institute of Biology, Leiden University, 2333 BE Leiden, The Netherlands

**Keywords:** morphotype, *Klebsiella*, experimental evolution, diversification, colony, rdar

## Abstract

The appearance of colony morphotypes is a signature of genetic diversification in evolving bacterial populations. Colony structure highly depends on the cell–cell interactions and polymer production that are adjusted during evolution in an environment that allows the development of spatial structures. Nucci and colleagues describe the emergence of a rough and dry morphotype of a noncapsulated *Klebsiella variicola* strain during a laboratory evolution study, resembling genetic changes observed in clinical isolates.

## Teaser

Commentary on the paper by Nucci et al that explains the genetic basis and fitness effects of specific mutations leading to rdar-like colony morphotypes in noncapsulated*Klebsiella variicola*.

Bacterial evolution in the laboratory is studied to understand the underlying genetic changes selected under specific experimental conditions. Intriguingly, certain mutations detected in the laboratory can also be observed in bacterial isolates from clinical or environmental settings, highlighting the relevance of laboratory evolution experiments. The first experimental evolution studies that exploited biofilm as laboratory model have immediately recognized the rapid emergence of colony morphotypes (Rainey and Travisano 1998, Poltak and Cooper 2011, Martin et al. 2016). Colony morphology diversification has been since also observed in host–microbe interactions (Pestrak et al. 2018, Blake et al. 2021, Nordgaard et al. 2022) and various *in-vitro* biofilm experiments (Kovács and Dragoš 2019, Xu et al. 2022). The colony morphotypes frequently differ in the ability to produce extracellular polymeric substances that constitute a matrix connecting the cells in a population. When bacteria are evolving in a spatially structured environment, the diversity in matrix production benefits the population, the mixture of evolved clones with variable levels of matrix secretion has higher population productivity or cell amount than the homogenous ancestor population. The difference in matrix production is due to mutations that either alter the regulation of the biosynthetic gene clusters, the function of metabolic pathways contributing to the synthesis of extracellular polymeric substances, or directly the synthesis machinery. These phenotypic changes influence cell–cell interactions. In addition to identifying the genetic changes, i.e. mechanistic understanding of affected processes, researchers are also intrigued by the underlying selection pressure and the impact of genetic differentiation on sociomicrobiology.

**Figure 1.**
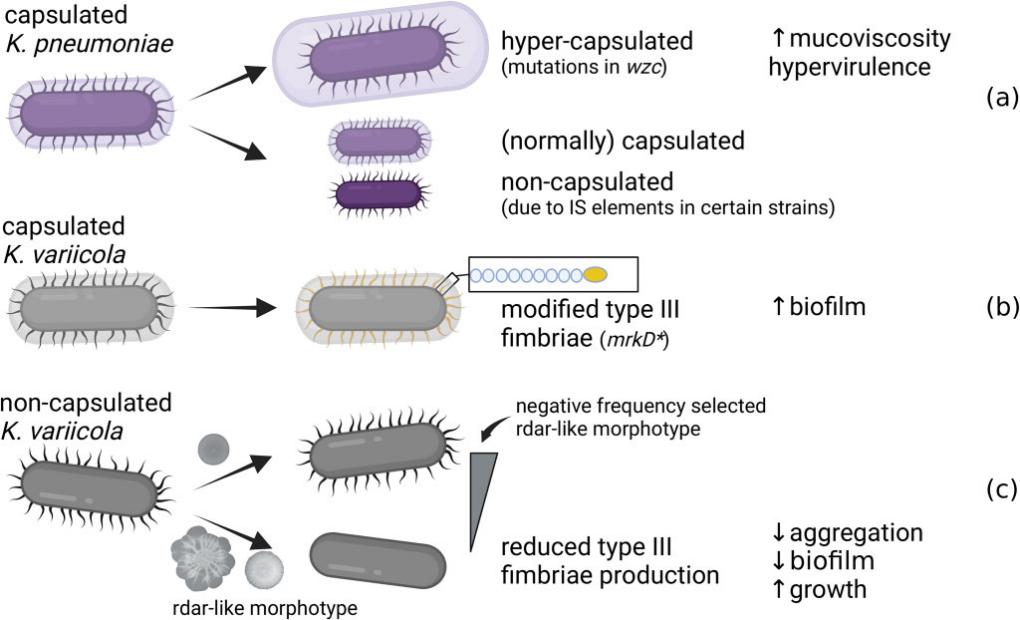
Depiction of diversification of *Klebsiella* spp. in experimental evolution studies: (a) the emergence of hyper-capsulated derivatives of *K. pneumoniae* (Nucci et al. 2022), (b) increased biofilm formation of capsulated *K. variicola* (Nucci et al. 2023b), and (c) rdar-like colony morphotypes of *K. variicola* (Nucci et al. 2023a). Figure was prepared on BioRender.com.

In their recent *MicroLife* publication, Nucci and colleagues identify the diversification of noncapsulated *Klebsiella variicola* strains evolved for 675 generations in environments with differing nutrient levels (Nucci et al. 2023a). After plating the independent populations from their evolution experiments, the researchers observed the emergence of a unique colony type resembling the rough and dry morphotype, i.e. rdar-like, observed in other *Enterobacteria* (Fig. 1). While the rdar-like morphotypes rely on the expression of curli or exopolysaccharides in *Enterobacteria, K. variicola* rdar-like colonies carry mutations in either the *mrkH* gene that encodes a transcriptional regulator controlling genes related to type 3 fimbriae production or the *nac* gene that codes a regulator for genes expressed in nitrogen-limited condition. Interestingly, *mrkH* is disrupted by an insertion sequence (IS) element in certain clones that display the rdar-like morphology. IS elements drive rapid loss of *K. pneumoniae* capsule production in experimentally evolved population (Nucci et al. 2022). IS elements have been previously implicated in the evolution of fuzzy spreader colony morphotypes of *Bacillus thuringiensis*, where an IS4-like element disrupts a gene encoding a guanylyltransferase, causing increased hydrophobicity and aggregation (Lin et al. 2022). In contrast to the enhanced aggregation of *B. thuringiensis* fuzzy morphotypes, *K. variicola* rdar-like derivatives display diminished aggregation (Nucci et al. 2023a). Nevertheless, the observed parallelism in the role of IS elements during the evolution of novel morphotypes highlights the impact of mobile genetic elements in rapid adaptation of bacteria, although it might be notable in a species- or strain-specific manner (Nucci et al. 2022, Hu et al. 2023).

The rdar-like clones of *K. variicola* display increased growth rate and fitness advantage compared with the ancestor (Nucci et al. 2023a). However, the fitness advantage is mostly prominent when the morphotype is in minority, demonstrating a negative frequency selection. Indeed, the morphotype frequency seems to increase during the experimental evolution, achieving up to 66% abundance in certain lineages, but displaying ∼16% frequency at the end of the study.

The rdar-like morphotype was detected after plating by Nucci and colleagues (2023a) when a noncapsulated *K. variicola* background was used, while colonies with distinct morphology were less apparent in capsulated strains that followed a different evolutionary path (Nucci et al. 2022). Subsequent experiments with strains carrying the mutant *mrkH* or *nac* alleles demonstrated that these mutations convey lower influence on cell–to–cell aggregation in the capsulated background and the fitness effects were only marginally larger in capsulated strains compared to noncapsulated strains. Historical contingency, where the existing mutations influence the subsequent adaptation path might explain the larger rdar-like morphotype frequency in noncapsulated *K. variicola*. This highlights the opportunity to discover novel evolutionary paths in strains lacking the most frequently mutated targets. Deleting the three most frequently mutated pathways in *Pseudomonas fluorescens* and subsequent experimental evolution in a static microcosm revealed 13 new mutational pathways that all result in wrinkly spreader colony morphotype (Lind et al. 2015). Interestingly, the fitness benefits of these novel mutations are also present in the ancestor background, although not as elevated as the common targeted paths, suggesting a hierarchical appearance of mutations driven by the superior fitness benefit (Lind et al. 2015). Furthermore, removal of exopolysaccharide synthesis in *B. subtilis* permits the evolution of clones with enhanced biofilm formation, explained by the production of novel, cysteine containing amyloid fibre variants (Dragoš et al. 2018). In contrast to the *P. fluorescens* example, the reconstituted *B. subtilis* strains with cysteine-encompassing amyloid fibres convey a disadvantage in the presence of the exopolysaccharide (Dragoš et al. 2018).

Finally, the work by Nucci et al. highlights the relevance of laboratory evolution for real-life scenarios. Detailed analysis of *K. pneumoniae* genomes revealed comparable IS element insertion in the *mrkH* gene of numerous clinical isolates. Isolates with interrupted *mrkH* genes were mostly originated from human host samples, including urine and blood as the main source (Nucci et al. 2023a). Yet, the frequency of these morphotypes is low in *Klebsiella* isolates, potentially explained by the negative frequency selection of these mutations. The detection of specific mutations or gene disruptions in natural populations further validate the relevance of experimental evolution studies in the laboratory settings as previously reported in *Klebsiella* (Nucci et al. 2023b) and in other species (Traverse et al. 2013, Lin et al. 2022).

The study of Nucci et al. (2023a) highlights the power of experimental evolution to understand genetic adaptation behind colony morphology diversification and connects the mutational landscape of laboratory-based experimental settings to natural bacterial populations.
